# Risk factors and incidence of unplanned re-operation after transumbilical single-hole laparoscopic appendectomy in children

**DOI:** 10.3389/fped.2025.1537897

**Published:** 2025-02-17

**Authors:** Yuanyuan Luo, Hong Zhang, Qiang Wu, Qianlong Li, Zhihua Ye, Jixiao Zeng, Xiaogang Xu

**Affiliations:** ^1^ Department of Gastrointestinal Surgery, Guangzhou Medical University Affiliated Women and Children’s Medical Center, Guangzhou, China; ^2^ Guangdong Provincial Key Laboratory of Research in Structural Birth Defect Disease, Guangzhou Medical University Affiliated Women and Children’s Medical Center, Guangzhou, China

**Keywords:** single-hole laparoscopic, acute appendicitis, children, appendectomy, unplanned re-operation, adhesive intestinal obstruction

## Abstract

**Purpose:**

This study aims to investigate the factors associated with unplanned re-operations (UR) following transumbilical single-hole laparoscopic appendectomy (TUSILA) in pediatric patients.

**Methods:**

We conducted a retrospective analysis of clinical data from children diagnosed with acute appendicitis (AA) who underwent TUSILA at our center between January 2020 and January 2024. All the operations were performed under single-port laparoscopy, including two methods of appendectomy, intra-TUSILA and extra-TUSILA. Patients were categorized into the UR and control groups to compare baseline characteristics, clinical data, postoperative management, and surgical outcomes.

**Results:**

The study included 188 patients (110 males and 78 females), with 4 (2.1%) in the UR group. Within the UR group, three cases (75%) necessitated re-operation due to adhesive intestinal obstruction, while one case (25%) was due to an appendiceal remnant fistula. The baseline characteristics, operation duration, intraoperative blood loss, surgeon experience, and postoperative fasting times showed no significant difference between the two groups (all *P* > 0.05). However, the incidences of procedures beyond standard TUSILA, lateral peritoneum lysis, appendiceal perforation, complicated appendicitis as confirmed by pathology, drainage tube placement, and the length of antibiotic duration were significantly higher in the UR group compared to the control group (all *P* < 0.05).

**Conclusion:**

A notable percentage of pediatric patients undergoing TUSILA experience UR, primarily due to adhesive ileus, with a substantial proportion potentially linked to surgical technical errors and postoperative management.

## 1 Introduction

Acute appendicitis (AA) is one of the most common emergency abdominal diseases in children, and surgical resection is the primary treatment for AA (1). With the advancement of minimally invasive surgery techniques, appendectomy has evolved from open surgery to laparoscopic appendectomy (2), which facilitates the reduction in postoperative pain, surgical incision infection, and postoperative recovery time. The conventional three-incision laparoscopic appendectomy (CTLA) has been recognized as a classic laparoscopic technique (3).

Since Esposito C. first reported a case of TUSILA in a child (4), the technique has become increasingly popular in clinical practice. The hidden incision induced by TUSILA has a cosmetic effect, and the cost of the technique is lower than CTLA, which makes the technique more suitable for children (5). Moreover, the safety and feasibility of TUSILA have been proven by many studies in both children and adults (6–9), leading to TUSILA being a reasonable alternative approach for CTLA. However, the abdominal cavity is undersized in pediatric patients, and surgeons may not fully visualize the structure. Although the reported safety is similar to CTLA, the TUSILA remains a technical difficulty for beginners. A study with 1948 consecutive patients found that the morbidity rate (7.0% vs. 6.5%, *p* *=* 0.795), readmission rate (2.1% vs. 1.3%, *P* *=* 0.414), and re-operation rate (0.8% vs. 0.8%, *p* = 0.348) were similar between learning and experienced periods. However, the rate of incisional hernia occurrence (0.6% vs. 0%, *P* = 0.066) tended to be more prominent in the learning period (9). Although not statistically significant, the tendency of cases with higher complication rates in the learning period should not be ignored due to the low complication rate.

Numerous adverse events can lead to unplanned re-operation (UR), which may extend hospital stays, increase healthcare costs, and worsen patient outcomes. Addressing UR is a critical concern in clinical practice, necessitating the identification of associated risk factors as a preliminary step. Despite the importance of this issue, there is a notable scarcity of research in this area. This study aims to retrospectively analyze data from pediatric patients diagnosed with AA at our center to identify factors associated with UR. The goal is to reduce the incidence of UR and enhance the quality of medical safety.

## 2 Materials and methods

### 2.1 Study population

Clinical data were collected and analyzed for all children who underwent TUSILA in the Women and Children's Medical Center Affiliated with Guangzhou Medical University from January 2020 to January 2024. All the patients received a 6-month follow-up after the first operation. Inclusion criteria: (1) preoperative clinical diagnosis of AA; (2) receiving TUSILA surgery; (3) postoperative pathology confirmed the diagnosis was AA. Exclusion criteria: (1) transfer to CTLA or open surgery during TUSILA; (2) chronic appendicitis was diagnosed before surgery, and more than one conservative treatment had been received; (3) previous history of abdominal surgery; (4) combined with other surgical conditions need to be treated at the same time; (5) combined with severe coagulation disorders, autoimmune defects, or other systemic diseases; (6) data missing or lost to follow-up.

The patients were divided into two groups according to whether UR was performed after surgery. Patients who underwent UR were included in the UR group, while those who underwent only appendectomy were included in the control group. This study was reviewed and approved by the Ethics Committee of the Women and Children Medical Center Affiliated with Guangzhou Medical University, and the child's guardian was informed and signed the informed consent.

### 2.2 Observation parameters

Baseline characteristics, including gender, age, body mass index (BMI), days from onset to surgery, and preoperative inflammatory markers, were recorded. Additionally, clinical data such as the timing of UR post-surgery and the primary causes of UR, initial appendectomy surgical details (surgical technique used, condition of the appendix during surgery, and pathological classification) along with postoperative management (the use of an indwelling drainage tube, the length of antibiotic duration and the postoperative fasting time) were collected for the study participants.

According to the grading system of the American Association for the Surgery of Trauma (AAST) (10), conditions were classified into two categories: uncomplicated appendicitis, which includes simple and suppurative appendicitis without perforation, and complex appendicitis, which includes gangrenous appendicitis, perforated appendicitis with abscess formation, and perforated appendicitis with diffuse peritonitis.

### 2.3 Surgical procedures

**Figure 1:**
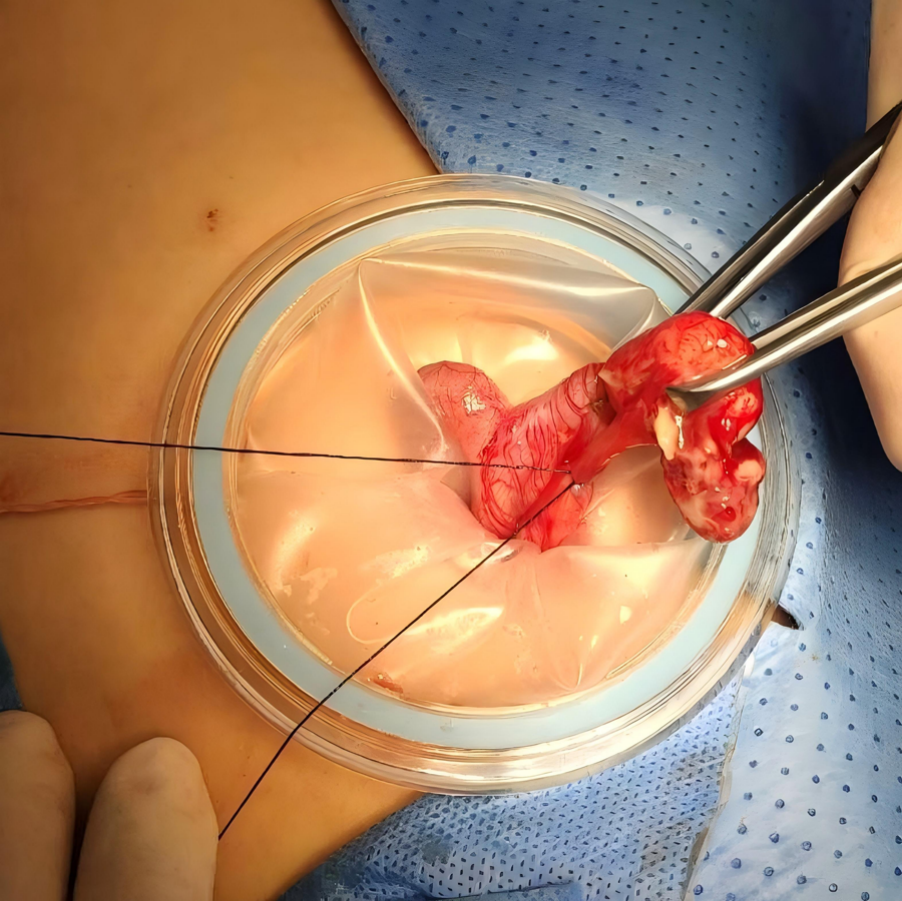
Appendectomy by extra-TUSILA.

(1) The multi-channel Trocar was inserted into the median Longitudinal transumbilical incision to establish pneumoperitoneum;
(2) Explore the abdominal cavity, isolate the adhesions, and look for the appendix;
(3) Appendectomy: A. Intra-TUSILA: removal of the appendix mesentery and appendix under laparoscopy *in vivo*, similar to CTLA; B. Extra-TUSILA: the appendix is clamped outside the umbilical incision and removed outside the body, similar to open surgery (Figure 1).

### 2.4 Statistical methods

SPSS 29 software was used for statistical analysis. First, a normality test was conducted for continuous variables of measurement data. $\bar{x}\pm s$ was used to describe those with normal distribution, and median (Q1, Q3) was used to describe those without normal distribution. The independent sample *t*-test was used when the normal distribution was obeyed, and the variance was homogeneous. The non-parametric test *U* test was used to compare groups. The paired *x*^2^ test was used to compare the enumeration data. *P* < 0.05 meant that the difference was statistically significant.

## 3 Results

### 3.1 The characteristics of the study population

After inclusion and exclusion criteria were adopted, 188 children received TUSILA during the study period. There were 10 cases (5.32%) in 2020, 12 cases (6.38%) in 2021, 51 cases (27.13%) in 2022, and 115 cases (61.17%) in 2023. Regarding appendectomy methods, 155 cases (82.45%) used Intra-TUSILA, and 33 cases (17.55%) used Extra-TUSILA.

Among the 188 patients, 30 cases (15.96%) developed postoperative complications, including 18 cases (9.56%) of incision infection, 8 cases of intestinal obstruction (4.26%), 5 cases of abdominal residual abscess (2.66%), of which 1 case was combined with incision infection. Among the 8 children with intestinal obstruction, 4 cases were improved by conservative treatment, 4 cases failed conservative treatment and finally received UR. In terms of annual incidence, UR was 0/10 (0%) in 2020, 0/12 (0%) in 2021, 1/51 (1.96%) in 2022 and 3/115 (2.61%) in 2023.

### 3.2 The therapeutic processes of children in the Ur group

**Figure 2:**
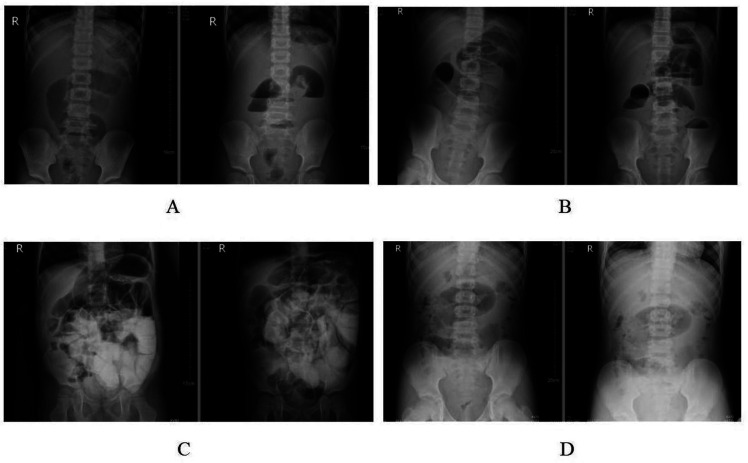
Preoperative x-rays of the abdomen in unplanned re-operation group. **(A)** Case 1; (**B)** case 2; **(C)** case 3; **(D)** case 4.

Case 1 was an 8 years old female. The first surgery was performed by the chief resident on 2022-1-10. The appendix perforation was found during the operation, the lateral peritoneum lysis was performed, and the operation was completed by extra-TUSILA. The postoperative pathology was suppurative appendicitis. She returned to the hospital due to “abdominal pain and vomiting for 4 days”. The strangulated ileus was suspected by abdominal x-ray (Figure 2A). UR was performed 14 days after the initial surgery. The ileocecal part and part of the ileum adhered to the pelvic cavity were observed.

Case 2 involved a 14-year-old male. The initial surgery, conducted by the chief resident on September 2023-9-16, did not result in appendix perforation. The lateral peritoneum underwent lysis via extra-TUSILA during the procedure, and no drainage tube was placed. The postoperative pathology revealed gangrenous appendicitis. He was readmitted eight days later with symptoms of abdominal pain and vomiting lasting 5 h and was diagnosed with adhesive ileus. Despite two days of conservative treatment, an abdominal x-ray showed no improvement. Considering the potential for strangulated ileus (Figure 2B), UR was performed 10 days post-surgery. During UR, adhesions were noted between the ileocecal part and the omentum to the right lower abdominal wall peritoneum.

Case 3 described a 4-year-old male. The chief resident performed the initial surgery on 2023-9-15, during which the appendix perforated and the lateral peritoneum underwent lysis via extra-TUSILA, with an intraoperative drainage tube placed. The postoperative pathology indicated suppurative appendicitis. Six days post-surgery, the patient exhibited abdominal pain, and an elevated infection index was noted. Intestinal obstruction was considered after seven days of ineffective conservative treatment, as indicated by an abdominal x-ray scan (Figure 2C). UR was necessary. Performed 13 days after surgery, the UR revealed that the hem-o-lok clamp on the appendix stump had detached, and extensive adhesions between the ileocecal part, the small intestine, and the right abdominal wall peritoneum were observed.

Case 4 was a 12-year-old male. The first operation was performed on 2023-6-18 by the chief resident. The appendix perforation occurred during the operation. The lateral peritoneum was not lysis, and the operation was completed intra-TUSILA. An intra-operative drainage tube was placed. The postoperative pathology was gangrenous appendicitis. After 118 days of surgery, he returned to the hospital due to “abdominal pain and vomiting for 1 day” and was considered to have adhesive ileus. After conservative treatment for 1-day, an abdominal x-ray scan showed no improvement, and the possibility of strangulated ileus was considered (Figure 2D). The UR was performed 119 days after surgery. During the operation, adhesion bands were observed between the omentum and the parietal peritoneum.

All patients in the UR group recovered well during the follow-up period and did not undergo the third operation.

### 3.3 Comparison of baseline characteristics between the two groups

There was no significant difference between the UR group and the control group in gender, age of operation, BMI, days of onset before surgery, last white blood cell, or C-reactive protein before surgery (*P* > 0.05) (Table 1).

**Table 1:** Comparison results of baseline characteristics between UR and control groups.

		UR group (*n* = 4)	Control group (*n* = 184)	*t*/*x*^2^	*P* value
Gender	Male	3 (75%)	107 (58.15%)	—^a^	0.643
	Female	1 (25%)	77 (41.85%)		
Age (years), median (IQR)		10 (5,13.5)	10 (7,12)	0	0.898
BMI, median (IQR)		14.41 (13.42,16.59)	15.54 (14.09,17.87)	0.995	0.297
Duration of symptoms, median (IQR)		1.5 (0.7,2.75)	1 (1,2)	0	0.891
WBC (×10^9^/L) before operation, median (IQR)		14.55 (11.23,18.33)	15.1 (11.8,18.38)	0.4	0.895
CRP (mg/L) before operation, median (IQR)		83.4 (25.65,139.7)	37.61 (12.68,78.33)	−24.45	0.258

BMI, body mass index; IQR, range interquartile; UR, unplanned re-operation.

^a^ Fisher exact test.

### 3.4 Comparison of initial operation parameters between the two groups

There was no significant difference between the UR and control groups in operation duration, intraoperative blood loss, or the surgeons' experience (*P* > 0.05). The percentages of extra-TUSILA, lateral peritoneum lysis during operation, appendix perforation during operation, and complicated appendicitis confirmed by pathology in the UR group were higher than those in the control group, and the differences were statistically significant (all *P* < 0.05) (Table 2).

**Table 2:** Comparison of operation data between the two groups.

		UR group (*n* = 4)	Control group (*n* = 184)	*P* value
Surgical types	Intra-abdominal, *n* (%)	1 (25%)	154 (83.7%)	**0.018**
	Extra–abdominal, *n* (%)	3 (75%)	30 (16.3%)	
Lateral peritoneum	With Lysis, *n* (%)	2 (50%)	8 (4.35%)	**0.015**
	Without Lysis, *n* (%)	2 (50%)	176 (95.65%)	
Appendix during surgery	Perforation, *n* (%)	3 (75%)	41 (22.28%)	**0.041**
	No perforation, *n* (%)	1 (25%)	143 (77.72%)	
Pathological	Simple, *n* (%)	0 (0%)	136 (73.91%)	**0.005**
	Complex, *n* (%)	4 (100%)	48 (26.09%)	
Experience of surgeon	Unexperienced, *n* (%)	4 (100%)	173 (94.02%)	>0.999
	Experienced, *n* (%)	0 (0%)	11 (5.98%)	
Operation time(min), median (IQR)		75 (61.25,111.3)	100 (80,130)	0.201
Intraoperative blood loss (ml), median (IQR)		2 (2,2)	2 (2,2)	0.943

IQR, range interquartile; UR, unplanned re-operation.

Bold values indicate *P* < 0.05.

### 3.5 Comparison of postoperative management between the two groups

The postoperative fasting time (*P* > 0.05) had no significant difference between the two groups, but the rate of drainage tube placement (50% vs. 8.15%, *p* = 0.042) and the length of antibiotic duration (8.5 vs. 6.0 days, *p* = 0.037) were statistically significant between the two groups (Table 3).

**Table 3:** Comparison of initial postoperative management between the two groups.

		UR group (*n* = 4)	Control group (*n* = 184)	*P* value
Drainage tube	Yes, *n* (%)	2 (50%)	15 (8.15%)	**0.042**
	No, *n* (%)	2 (50%)	169 (91.85%)	
Length of antibiotic duration (days), median (IQR)		8.5 (7,27.5)	6 (5,8)	**0.037**
Postoperative fasting time (days), median (IQR)		1.5 (1,3.5)	1 (1,2)	0.408

IQR, range interquartile; UR, unplanned re-operation.

Bold values indicate *P* < 0.05.

## 4 Discussion

AA is one of the most common acute abdominal diseases in children, and appendicectomy mainly reflects the basic skill level of general surgeons. However, the UR rate for pediatric abdominal surgery was as high as 0.8%–7%, with appendicectomy ranked first (11, 12). UR is widely used as an indicator to track surgical quality improvement as UR is frequently the consequence of complications from the initial procedure. It is a crucial tool for safety audits and reviews in surgical departments and hospitals. The information may also be used as selection criteria for other clinics and patients to look for a suitable medical institution (13, 14). Monitoring and improving the incidence of UR is crucial for the hospital administrator.

With advancements in laparoscopic techniques, TUSILA is increasingly utilized in clinical practice. A meta-analysis concluded that the efficacy and safety of TUSILA were acceptable compared to the traditional CTLA (15). However, data on the rate of UR following TUSILA remains scarce. Since the initial TUSILA procedure at our hospital, a UR incidence of 2.1% has been recorded over four years. An upward trend in UR rates has been noted annually with the growing number of TUSILA cases. This increase may be attributed to the learning curve associated with the technique. TUSILA requires the insertion of devices through a single channel in the umbilical cord, which disrupts the optimal laparoscopic working triangle, leading to the “chopstick effect.” Moreover, the surgeon and the assistant must maintain awkward ergonomic positions for extended periods, complicating the procedure further. Compared to traditional laparoscopic surgery, TUSILA demands a longer and more challenging learning curve (16). Kim et al. reported that surgeons typically acquire essential surgical competencies in TUSILA after 30 procedures and achieve proficiency after 90 operations (17). As the number of cases increases, many surgeons are likely in the early stages of their experience, contributing to the higher UR rates.

Previous studies showed that patient characteristics were poor in predicting UR, and surgical technical errors were the primary cause of UR, accounting for 57.9% (11, 18). Our study showed no significant differences in baseline characteristics between the UR and the control groups. Of the 4 children who received UR, 3 were due to adhesive intestinal obstruction, and 1 was due to appendiceal remnant fistula. All of the complications contributed to the second surgery. We can also conclude that the primary cause of UR was surgical technical error.

In the same case of TUSILA, different surgeons have different details. In terms of dealing with the appendix, there are different surgical methods, mainly including (1) intra-TUSILA, which removes the mesangium and appendix by using laparoscopic instruments *in vivo*, similar to CTLA (19–21); (2) extra-TUSILA, mesangium, and appendix are clamped outside the umbilical incision by using laparoscopic instruments and removed combining using routine surgical instruments *in vitro*, similar to open surgery (22, 23). Most of the previous studies did not distinguish the two methods or only limited the discussion to one of the surgical methods. However, we believe that the two surgical methods have essential differences in operation and technical difficulty. In general, intra-TUSILA requires a higher level of surgical skill, which may be difficult for younger surgeons to achieve. The abdominal wall of children is flexible, and the distance from the cecum to the umbilicus is short, which makes it easy for the appendix to be placed outside the umbilical cord (24). Extra-TUSILA capitalizes on specific anatomical characteristics of children by processing the appendix externally, simplifying operations through a single incision, and significantly shortening the learning curve, rapidly gaining popularity in pediatric surgery. However, Extra-TUSILA is not suitable for all cases. Challenges arise when the appendix is severely swollen, perforated, or adherent, when the cecum is fixed in older children or when retroperitoneal fixation occurs due to anatomical variations. These conditions increase the risk of iatrogenic perforation, tearing, and intraperitoneal spillage as the appendix is forcibly pulled to the umbilical cord (25). In this study, the use of Extra-TUSILA in the UR group was significantly more prevalent than in the control group (75% vs. 16.3%). Additionally, rates of appendix perforation (75% vs. 22.28%), proportions of complicated appendicitis confirmed by postoperative pathology (100% vs. 26.09%), and incidences of lateral peritoneum lysis (50% vs. 4.35%) were all markedly higher in the UR group. These conditions complicate the surgical environment, exacerbate inflammation, and restrict the appendix, elevating the risk of postoperative adhesive ileus (26), and intra-TUSILA is suitable for these cases. However, such cases are often admitted as emergencies, where the chief resident often does emergency surgery, and when younger doctors have not yet overcome the learning curve, the simpler extra-TUSILA is the preferred procedure. To place the appendix outside, it is necessary to loosen the lateral peritoneum, and additional peritoneal injury will lead to the destruction of peritoneal mesothelial cells, which will trigger a series of cascade reactions such as coagulation, inflammation, and fibrinolysis and eventually lead to fibrin exudation and abnormal tissue repair. As a result, adhesive intestinal obstruction can sometimes occur, eventually leading to UR (27, 28). Therefore, the adhesive ileus and UR risk is higher in these cases. In addition, one case in the UR group was caused by an appendix remnant fistula, which should also be classified as a surgical technical error. The appendix stump was not fully sutured. Generally, the above surgical techniques and intraoperative decision-making factors are involved in the occurrence of UR. Therefore, young surgeons need to improve their surgical skills and make the right surgical decisions according to the intraoperative situation to ensure the safety of TUSILA.

In addition to surgical technical errors, postoperative management mistakes were the second most common cause, accounting for approximately one-fifth of all cases (12). In this study, the proportion of postoperative drainage tube placement in the UR group was significantly higher than in the control group (50% vs. 8.15%). Although postoperative drainage tube placement facilitates the timely discharge of blood and fluid, thus reducing the incidence of infection, it can also serve as a foreign body that promotes fibrin exudation and increases the risk of adhesive intestinal obstruction (29). Furthermore, a drainage tube may delay the postoperative mobility of children and slow the recovery of intestinal function. Therefore, careful evaluation of the necessity for drainage tube placement during surgery, timely assessment of post-surgery condition, and prompt removal of the drainage tube are critical to reducing the risk of intestinal adhesion and UR. Moreover, the duration of antibiotic use during the perioperative period of the initial surgery was significantly longer in the UR group (8.5 days vs. 6 days). The results revealed that patients in the UR group experienced more severe systemic inflammation, and the risk of long-term intestinal adhesion was higher. Therefore, for patients with severe systemic inflammation, close observation after surgery is urgently needed, and timely UR may improve the prognosis. In addition, in our center, the concept of enhanced recovery after surgery (ERAS) in postoperative management was introduced several years ago, and most patients can resume their diet within 1–2 days after surgery. Our study showed that the postoperative fasting time after surgery was comparable between the two groups, which indicated that ERAS was safe and feasible after pediatric TUSILA.

This study has several limitations. The sample size was small, and the data was collected from a single institution, which precludes the possibility of conducting logistic regression analysis and introduces significant selection and statistical biases. Future multi-center studies are needed to analyze risk factors and establish a risk prediction model.

The incidence of UR in children following TUSILA is approximately 2%. Factors such as procedures additional to TUSILA, lysis of the lateral peritoneum, appendix perforation, drainage tube placement, and complicated appendicitis confirmed by pathology may be risk factors for UR. Surgeons should accurately assess the appendix's condition during surgery, choose the appropriate surgical technique, and optimize both intraoperative and postoperative management to significantly reduce the risk of UR.

## Data Availability

The raw data supporting the conclusions of this article will be made available by the authors, without undue reservation.
